# Magneto-plasmonic “switch” device for magnetic field detection

**DOI:** 10.1515/nanoph-2024-0136

**Published:** 2024-06-06

**Authors:** Laure Bsawmaii, Pascal Giraud, Gerges El Haber, Lukas Halagacka, Jean-Pierre Chatelon, Damien Jamon, Yves Jourlin, François Royer

**Affiliations:** 131842 Université Jean Monnet Saint Etienne, CNRS, Institut d’optique Graduate School, Laboratoire Hubert Curien UMR 5516, F-42023 Saint-Etienne, France; Faculty of Materials Science and Technology, VSB – Technical University of Ostrava, 17 Listopadu 15, 708 00 Ostrava-Poruba, Czech Republic; Nanotechnology Centre, CEET, VSB – Technical University of Ostrava, 17. Listopadu 15, 70800 Ostrava, Czech Republic

**Keywords:** plasmonic, magnetic field sensors, magneto-optics

## Abstract

This paper introduces a novel class of low-loss and cost-effective optical planar structures tailored for magnetic detection applications. These structures represent unconventional magneto-plasmonic devices specifically optimized for an ‘optical switch’ configuration. The structure consists of a 1D deep sinusoidal gold grating covered by a thin cobalt layer. In this unique arrangement, the excited plasmon induces a high-contrast switching phenomenon between the reflected free space intensity of specular (0th) and −1st diffracted orders, sensitive to any transverse magnetic fields applied to the cobalt layer. The use of these two distinct diffracted orders induces differential measurements, effectively mitigating common drift and perturbations. This innovative approach results in an enhanced detection sensitivity, showcasing the potential of these structures for advanced magnetic field sensing applications.

## 1 Introduction

Surface plasmon resonance (SPR) sensors [1], [2], [3] are commonly used for the detection and identification of various dielectric media, including gases [4] (H_2_S, a flammable and toxic gas) and fluids [2]. These devices are particularly valuable in analyzing bio-samples, e.g. water solutions, urine, and proteins, making them a versatile tool in the field of biosensing and analytical chemistry. These sensors operate through the resonant excitation of surface plasmon polaritons (SPPs) excited at the interface between metal and probatic dielectric media [5]. The wave vector relies on the optical properties of both the metal and the dielectric medium. Consequently, any variation in the refractive index, of the probed dielectric medium induces a shift in the resonance of SPPs [6]. Thus, the measurement of the shift in the reflectivity spectrum serves as the operating principle of SPR sensors.

SPR sensors have gained considerable attention due to their potential benefits, which include label-free, real-time, and rapid detection capabilities [7], [8].

The combination of SPR with a magneto-optical (MO) material holds great promise for applications in magnetic field sensing and in bio-sensing [1], [6], [9]. MO materials [10] are substances that can modify their interaction with light (polararization rotation and phase shift) when magnetized [11], [12]. In the context of SPPs guided at a metallic/dielectric interface, where one of the two media exhibits MO properties and is subjected to a transverse magnetic field (oriented in *y* direction and perpendicular to the incident plane, *x* − *z* plane), the propagation constant of SPPs evolves according to the following equation [13]:

(1) $$\beta_{\text{SPPs}}=k_{0}\sqrt{\frac{\epsilon_{d}.\epsilon_{m}}{\epsilon_{d}+\epsilon_{m}}}\left(1+\frac{\epsilon_{xz}}{\sigma }\right)$$

where,

$$\sigma =\sqrt{-\epsilon_{m}\epsilon_{d}}\left(1-\frac{\epsilon_{d}^{2}}{\epsilon_{m}^{2}}\right)$$

Here, *ϵ*_*xz*_ is the off-diagonal term of the dielectric tensor of the MO material, proportional to the magnetization. *ϵ*_*d*_ and *ϵ*_*m*_ are, respectively, the permittivity of the dielectric and magnetic film, and *k*_0_ is the wavevector. It is thus evident that when *ϵ*_*xz*_ equals zero (Magnetization *M* = 0), Eq. (1) simplifies to the standard propagation constant of SPPs [5]. However, when *ϵ*_*xz*_ is not equal to zero (*M* ≠ 0), the propagation constant of SPPs undergoes a non-reciprocal modification when the magnetization is reversed through the applied magnetic field.

Optical fiber sensors based on SPR and coated with ferrofluid [14], [15], liquid containing magnetic solid particles, have attracted researchers’ attention. This configuration exhibits sensitivity to both temperature and magnetic fields, allowing for the concurrent measurement of these two parameters. Nevertheless, the practical application of this technology has faced limitations due to the instability of the magneto-fluid, hindering its further development. Additionally, these sensors are challenged by complex fabrication processes, potential high costs and alignment sensitivity.

Recently, a MO-SPR structure based on Au/Co/Au thin films demonstrated a promising approach for detecting magnetic fields [16]. The results shows that the device exhibits a good sensitivity with small Co thicknesses (5 nm–12 nm), fast response time (around 100 ns), and long-term stability. However, these structures suffer from low optical quality resonances (reflection dip) caused by the high absorption of plasmonic oscillations, which limits their practical applications.

This paper proposes low-loss and cost-effective optical planar structures that may hold great promise for magnetic detection applications. They are unconventional magneto-plasmonic devices optimized for a so-called configuration “optical switch” [17]. The structure consists of a 1D deep sinusoidal gold grating covered by a thin cobalt layer. In this configuration, the excited plasmon triggers a high-contrast switching between the reflected free space intensity of 0th and −1st diffracted orders, upon any transverse magnetic field applied to the cobalt layer. Hence, the monitored specular and −1st diffracted order enable differential measurements.

The novelty in this study lies on using precisely designed diffraction magnetoplasmonic grating for differential measurements. The key benefits are insensitivity on the source light fluctuation, fast time-response, and low optical losses ensuring promotion of the signal-to-noise ratio.

To our knowledge, this combination of features has not been employed in magnetic field sensors previously. Hence, this work offers several significant advantages. One of the key advancements of our study is the use of these two distinct diffracted orders (specular/0th and −1st) enabling differential measurements of two reflected light intensities. Traditional magneto-plasmonics [4], [9] are typically based on detection of a single reflection deep, which is related to low-intensity signal detection. Our presented differential approach effectively eliminates common drift and perturbations, resulting in enhanced detection sensitivity and accuracy.

Additionally, the studied structure exhibits lower losses compared to other magneto-plasmonic devices proposed in the literature. Traditional magneto-plasmonic devices often suffer from low-optical quality resonances [6], primarily caused by the high absorption of plasmonic oscillations. By minimizing these losses, the structure demonstrates improved performance and expanded practical applications.

Furthermore, the simplicity of the studied structure holds promise for magnetic field sensing applications. We are convinced that this approach represents a breakthrough in processing efficient magneto-optical sensors on various types of substrates, including large-scale or non-conventional surfaces.

This paper is organized as follows: firstly, the structure design is introduced, outlining the principles of the optical switch, followed by a presentation of the optimized structure parameters. Secondly, the Materials and Methods section illustrates the fabrication steps of the structure and details the experimental measurement setup. Finally, the Results and Discussions are presented, culminating in a comprehensive Conclusion summarizing the findings.

## 2 Magneto-plasmonic “switch”

**Figure 1:**
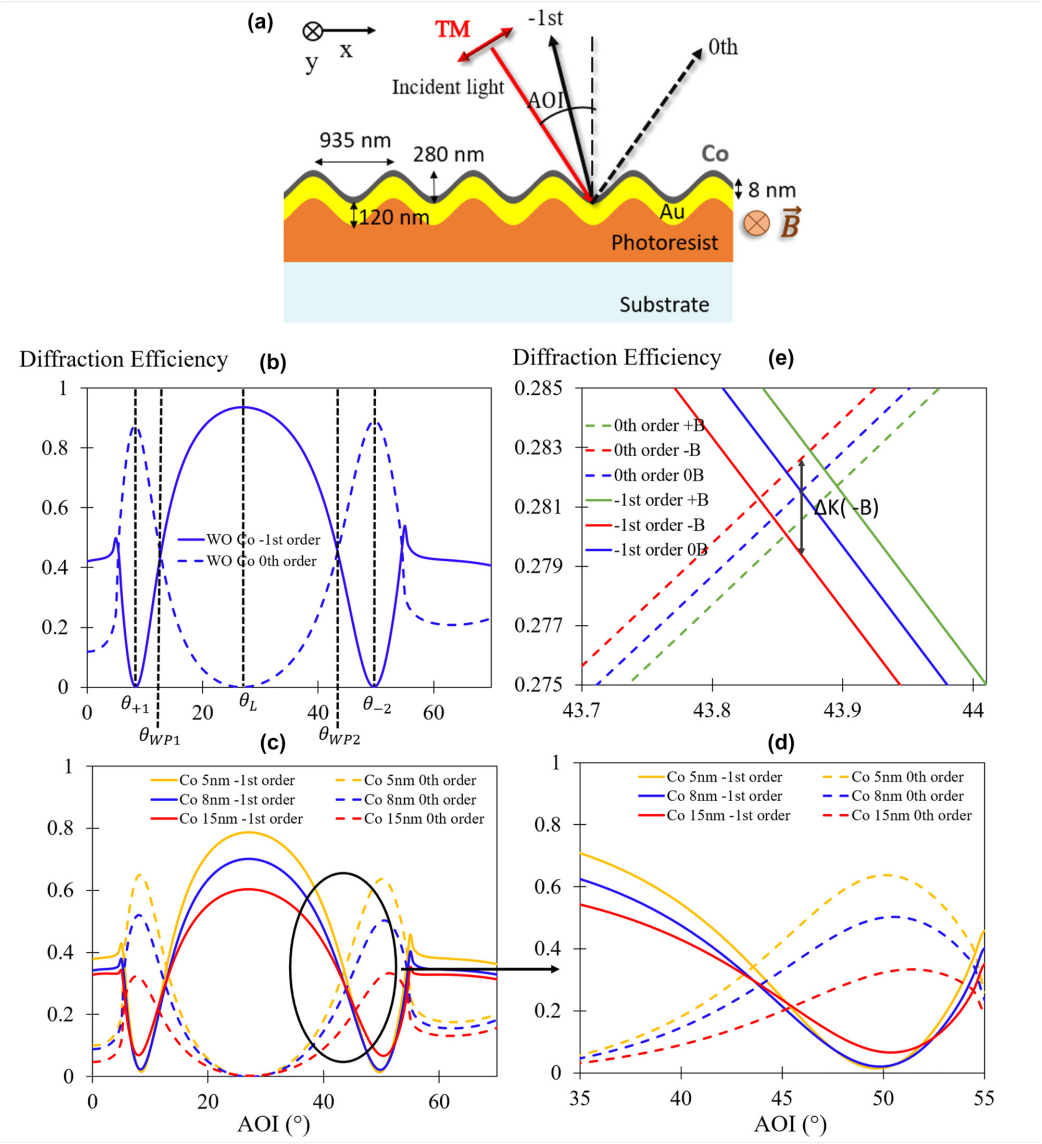
Numerical study of the magneto-plasmonic switch effect. (a) Schematic illustration of the magneto-plasmonic structure with TM-polarized incident light. Simulated angular spectra of diffraction efficiency for 0th and −1st diffraction orders for grating: (b) without (WO) Co, (c) and (d) with different Co thicknesses. (e) Simulated angular spectra of diffraction efficiency for 0th and −1st diffraction orders for grating with 8 nm of Co under an applied magnetic field. Here, we are referring to a saturating B, inducing the full magnetic alignment of M of Co along the *y* direction.

This section initially describes the optical switch phenomenon, followed by a numerical study of optical and MO behaviors of the Co–Au grating. The device under consideration is schematically drawn on Figure 1(a).

### 2.1 SPR sensor based on plasmon-triggered switching

The switch effect is a plasmon-based phenomenon that employs a metallic sinusoidal diffraction grating where both the 0th and −1st propagative diffractions orders are involved (see Figure 1(b)). This effect was first evidenced by Sauvage-Vincent et al. [18], [19], and fully analyzed in [17]. Comparatively to classical plasmonic resonant devices where a dip in energy is sought, the “switch” device induces a quasi-lossless interaction mechanism between the involved modes and the diffractive orders, allowing for a differential measurement of the intensities of the two reflective orders, making the system insensitive to variations of the optical source. The measurement, achieved at a fixed angle and fixed wavelength, is then more robust, cost, and size-efficient.

To obtain a plasmon-triggered switch configuration, the period and the grating depth must be tuned. The former is chosen in order to insure the free-space propagation of both the 0th and the −1st diffraction orders in reflection. The depth should be large enough and precisely chosen to enable the switch effect [17], which manifests as follows: when varying the incident angle, the intensities of the two reflective orders varies from a maximal value to zero in an opposite manner, i.e. when 0th is maximum, −1st is zero and *vice-versa*. Figure 1(b) illustrates the simulated angular switch pattern for a gold grating with a period of 935 nm and a depth of 280 nm under an incident TM (Transverse Magnetic)-polarized plane wave light of wavelength *λ* = 850 nm. At the first peak of the 0th order (incident angle equal to *θ*_+1_), a co-propagative plasmon mode is excited through the evanescent +1st diffraction order. At the second one (*θ*_−2_), a counter-propagative plasmon mode is excited through the evanescent −2nd diffraction order. The large interaction efficiency of such deep grating induces that the required distance for the plasmon mode to be re-irradiated in the 0th order is shorter than the required distance to be attenuated [17]. Thus, the mode does not have time to get fully attenuated, and a quasi-lossless reflection is observed for the 0th order at these specific angles. In an opposite manner, a dip in the 0th order and a peak in the −1st order are simultaneously reached for the deep metallic grating [17], [20] at *θ*_*L*_, corresponding to the Littrow angle where the −1st order is diffracted in the same direction as the incident light source. Finally, the “switch” configuration allows obtaining two points of interest in the angular pattern, so-called working points, where 0th and the −1st orders intensity curves cross each other at *θ*_*WP*1_ and *θ*_*WP*2_.

In a conventional application, the device operates at one of these working points to detect the modification of the ambient medium. Indeed any change of its surrounding permittivity modifies the plasmon propagation constant (see Eq. (1)) and thus the resonant coupling condition of the modes. It leads to an angular shift of the pattern, which results as opposite variations of the 0th and the −1st orders intensities at the working points *θ*_*WP*1_ or *θ*_*WP*2_. Then, achieving a differential measurement in terms of optical intensities at one of these working points allows triggering any modification of the ambient medium, resulting in a non-zero difference. It has been shown that such differential sensing configuration, embedded in a fluidic cell, induces a limit of detection of 10^−6^ RIU of the refractive index of the liquid [21].

In the following sub-section, the magneto-plasmonic switch device will be analyzed.

### 2.2 Numerical study of plasmon-triggered switching for magnetic field detection

To make the device sensitive to magnetic fields, a thin layer of cobalt has been deposited on the sinusoidal gold grating (see Figure 1). Due to the MO character of the cobalt, the plasmon mode propagation constant varies as a function of the magnetization of the film as illustrated through equation (1), leading to a switch effect that depends on the applied magnetic field.

The device reported on Figure 1 is made of a deep grating of 280 nm depth and a period of 935 nm. These optimized grating parameters were obtained through MC Grating software [22]. Simulations are based on the Chandezon method [23], which is well-suited for gratings with a sinusoidal shape. The main idea of the method is to simplify the continuity condition at the grating surface by making it planar, thanks to a modification of the space coordinate. Compared to the RCWA method [24], the Chandezon method is also more appropriate for plasmonic simulation, since the TM polarization induces convergence issues for RCWA. On the other hand, the RCWA methods are more suitable for materials with general anisotropy, especially of anti-symmetric type, i.e. magnetooptics. This is a consequence of the fact, that continuity of electromagnetic fields is achieved via proper factorization approach instead of coordinate and material transformations for planarization. To obtain a switch configuration, the grating was designed on an infinite gold grating under an incident light of 850 nm in TM polarization. The depth and the grating period were the two tuning parameters. The depth affects the diffraction efficiency, while the grating period influences the width of the switch eye. Once a first switch configuration is obtained where the both orders cross, an optimization of the 0th order is conducted to set it at a minimum value by tuning the grating depth, generally under 10^−6^. Since our experimental setup does not allow us to work with a second crossing point under 35°, an optimization is conducted on the second crossing point to set it at 45° by dichotomy by tuning the grating period. Thus, the founded optimized parameters for an infinite gold grating under an incident light of 850 nm in TM polarization are a depth of 280 nm and a grating period of 935 nm. The optimized parameters were only conducted for an infinite gold grating, which does not include the Co layer. Since we only work with thin Co layers at the nanometer scale, the thin layer induces a general attenuation of the diffraction efficiency and a slight narrowing of the switch eye, always up to 40°. Therefore, our optimized gold grating offers us the flexibility to work with different Co layers without compromising the switch configuration, as long as the layer is thin.

The optical and MO response presented on Figure 1(c)–(e) were calculated using in-house developed RCWA code for 1D gratings and materials with general anisotropy. In order to achieve good agreement with numerical data provided by Chandezon method, the number of structure slices and number of Fourier harmonics used for field expansion were optimized.

In Figure 1(c) and (d), the simulated diffraction efficiency angular curves for various Co thicknesses (5 nm, 8 nm, and 15 nm) are illustrated for 0th and −1st diffracted orders. These curves reveal the presence of a crossing point for different thicknesses, with the noteworthy observation that this crossing point is shifted. This shift can be attributed to the fact that as the Co thickness increases, the peak values of the curves decrease, while the dip values increase, as shown in Figure 1(c) and (d), consequently decreasing the switching slope. This behavior can be attributed to the absorption characteristics of the Co material.

Figure 1(e) illustrates the simulated diffraction efficiency spectra (for 0th and −1st orders) for the structure with 8 nm of Co, when a transverse magnetic field is applied (perpendicular to the incident plane) for both direction. As observed in this figure, there is an angular shift in the position of the crossing point (blue curve at zero field) when a transverse magnetic field is applied (red and green curves). As explained before, this angular shift is linked to the MO effect of the Co layer and explained through Eq. (1), where the propagation constant of SPPs is modified with an applied transverse magnetic field. Notably in Figure 1(e), this shift is non-reciprocal, meaning it is opposed when the direction of the magnetic field is altered (±*B*), which is consistent with Eq. (1). The change in the diffraction efficiency (Δ*K*) at the zero field crossing angle point can be calculated to be approximately 1 % for +*B*_sat_ or −*B*_sat_. Here, we are referring to a saturating B, inducing the full magnetic alignment of M of Co along the *y* direction, resulting in an off-diagonal element of Co equal to: *ϵ*_*xz*_ = 2 + 0.7*i*. After studying numerically these structures, the following sections will showcase the experimental demonstration for various Co thicknesses, following the detailed description of the fabrication process and the experimental setup.

## 3 Materials and Methods

### 3.1 Fabrication process

**Figure 2:**
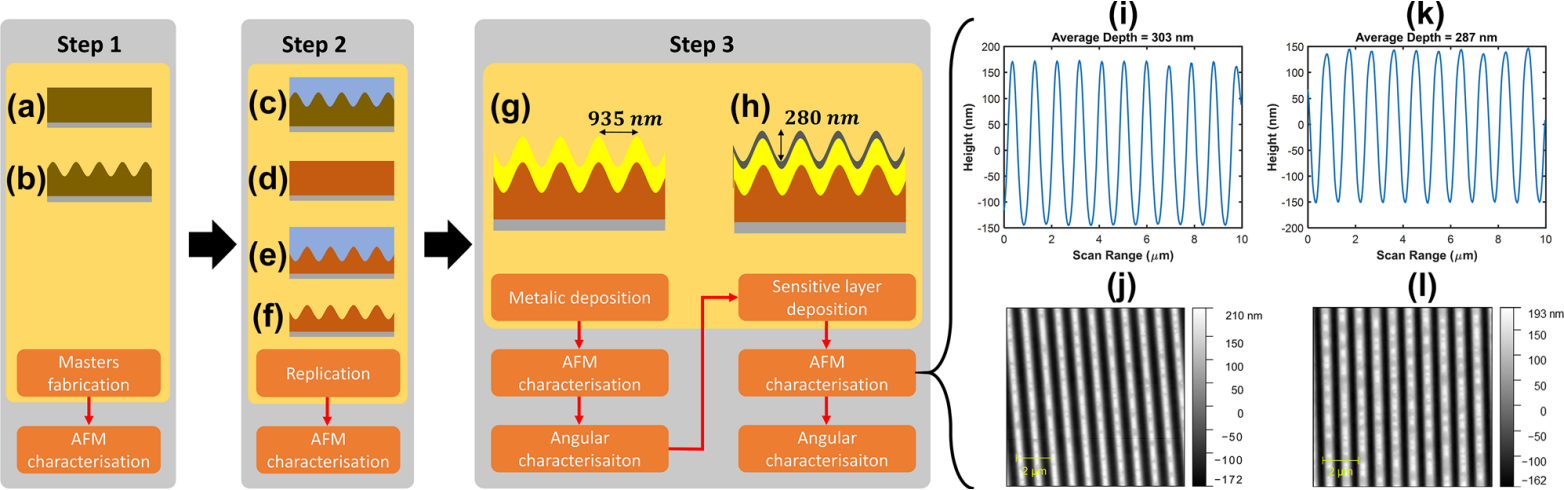
Fabrication steps of the structure with: (a) photoresist layer deposition on a glass substrate, (b) photoresist layer grating master through LIL process, (c) shape copy of the master, (d) sol gel layer deposition on a glass substrate, (e) embossing of the sol–gel layer by the mold, (f) final replica structure after layer stabilization under UV insolation, (g) chromium and gold deposition on the replica, (h) final structure after the sensitive Co layer deposition, (i) height grating profile for the structure after 120 nm gold deposition, (j) AFM grating profile after 8 nm of Co deposition, (k) height grating profile after 8 nm of Co deposition, (j) AFM grating profile after 8 nm of Co deposition.

As depicted in Figure 2, photoresist layers deposited on glass substrates through a spin-coating process were patterned with a sinusoidal shape using laser interference lithography (LIL), achieving a period of 935 nm and a depth of up to 300 nm. Among the batch of samples, only some were usable, and these were subsequently replicated by NAPA company using the nano imprint lithography (NIL) method. A loss in depth of between 5 and 30 % is generally observed, but the initially overshooted depth on the master samples allows for achieving the targeted depth of 280 nm on the replicas with a tolerance of 5 %.

Next, the metallization of replicas is performed using two thermal evaporation processes. The first one is for the thin bonding layer of 6 nm chromium, and the next one is for the gold deposition of 120 nm thickness (Figure 2(g)). Here, the thickness value guarantees that orders are diffracted in reflection. Also, the computed penetration depth of the plasmon’s evanescent field in the gold metal is around 37 nm, and the 120 nm gold thickness, up to three times the penetration depth, ensures that the plasmon’s evanescent field in the gold metal does not extend out and interact with the ambient medium. Next, an optical characterization is performed by measuring the efficiencies of the 0th and the −1st reflected orders versus the incident angle with an optical setup. A laser diode with an 850 nm wavelength in TM configuration and a photodiode are mounted on two mechanical arms connected to two rotational stages. The sample is set on a holder placed at the rotation center of the two rotational stages, which can be adjusted in the three spatial translations and rotations. These degrees of freedom allow the setup to be adjusted so that the photodiode, the laser diode, the sample, the 0th, and the −1st orders are in the same incidence plane. The angular spectrum is then checked to verify the switch configuration and the crossing points at two particular angles. Figure 4(a) shows a typical angular spectrum of the switch configuration, where the two crossing points are located at 19° and 37°.

Finally, a few nanometers of cobalt are deposited as a sensitive layer using the DC-magnetron sputtering method. The deposition conditions include a base pressure of 1 × 10^−7^ mBar, deposition pressure (Argon) of 1.2 × 10^−2^ mBar, a constant power of 50 W, and a deposition rate of around 0.138 nm/s (Figure 2(h)). As shown in Figure 2(i)–(l), the cobalt deposition did not induce a structural modification. The depth difference of a few nanometers between the gold-coated structure and the added 8 nm of cobalt is not significant and is mainly due to a slight inhomogeneity of the grating, since the AFM measurement was not performed exactly at the same point. Fourier spectra computed on the measured profile also indicate that the sinusoidal profile is maintained after the cobalt deposition, as the number of harmonics and the HWHM (half width at half maximum) of the fundamental frequency remained unchanged. Then, an angular characterization is carried out again to observe the effect of the sensitive layer on the switch curve (see Figure 4(a)). The presence of the switch curve pattern and the two crossing points allows to validate the structure for measurements under magnetic fields.

### 3.2 MO measurements setup

**Figure 3:**
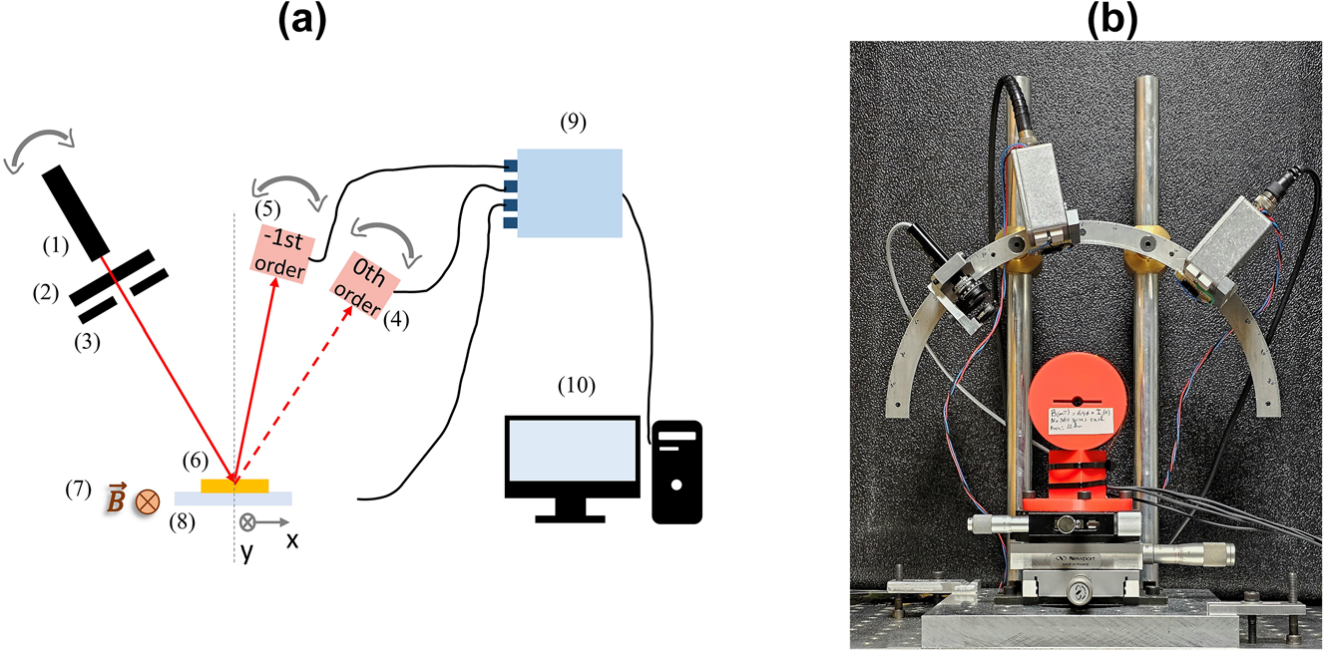
Setup used to perform the MO measurements. (a) It consists of an 850 nm wavelength laser diode (1), a polarizer (2), an aperture (3), and two photodiodes (4) and (5) to measure the power in the −1st (dashed red line) and the 0th (solid red line) orders diffracted from the grating (6) placed between Helmholtz coils generating a transverse magnetic field (7) and mounted on a *xy* stage (8). A DAQ (data acquisition) device (9) and a computer (10) were used to record the measurements. (b) Photograph illustrating the MO experimental setup.

The measurements setup (Figure 3) consists of a laser diode (*λ*_0_ = 850 nm) followed by a polarizer used to fix the incident polarization to TM and an aperture to remove background light. The grating is placed in the air gap of Helmholtz coils mounted on a *xy* stage and generating a magnetic field oriented in *y* direction, perpendicularly to the incident plane *x* − *z*. The magnetic field’s amplitude can be adjusted within the range of [−40 mT; +40 mT] using a 4-quadrant linear amplifier. Two Si-based photodiodes are employed to analyze the reflected light from the grating. Both photodiodes are used to measure the power of the 0th and −1st diffraction orders. The laser diode and photodiodes are situated on a manually rotating stage to fix the incident angle (crossing point) and detect the corresponding diffracted angles.

The photodiode currents are converted to voltage through a transimpedance amplifier circuit, resulting in an output voltage proportional to the incident optical signal. Finally, an analog-to-digital converter (ADC) and Labview software are used to acquire voltage data from the photodiodes, and from the amplifier which monitor the magnetic field amplitude.

**Figure 4:**
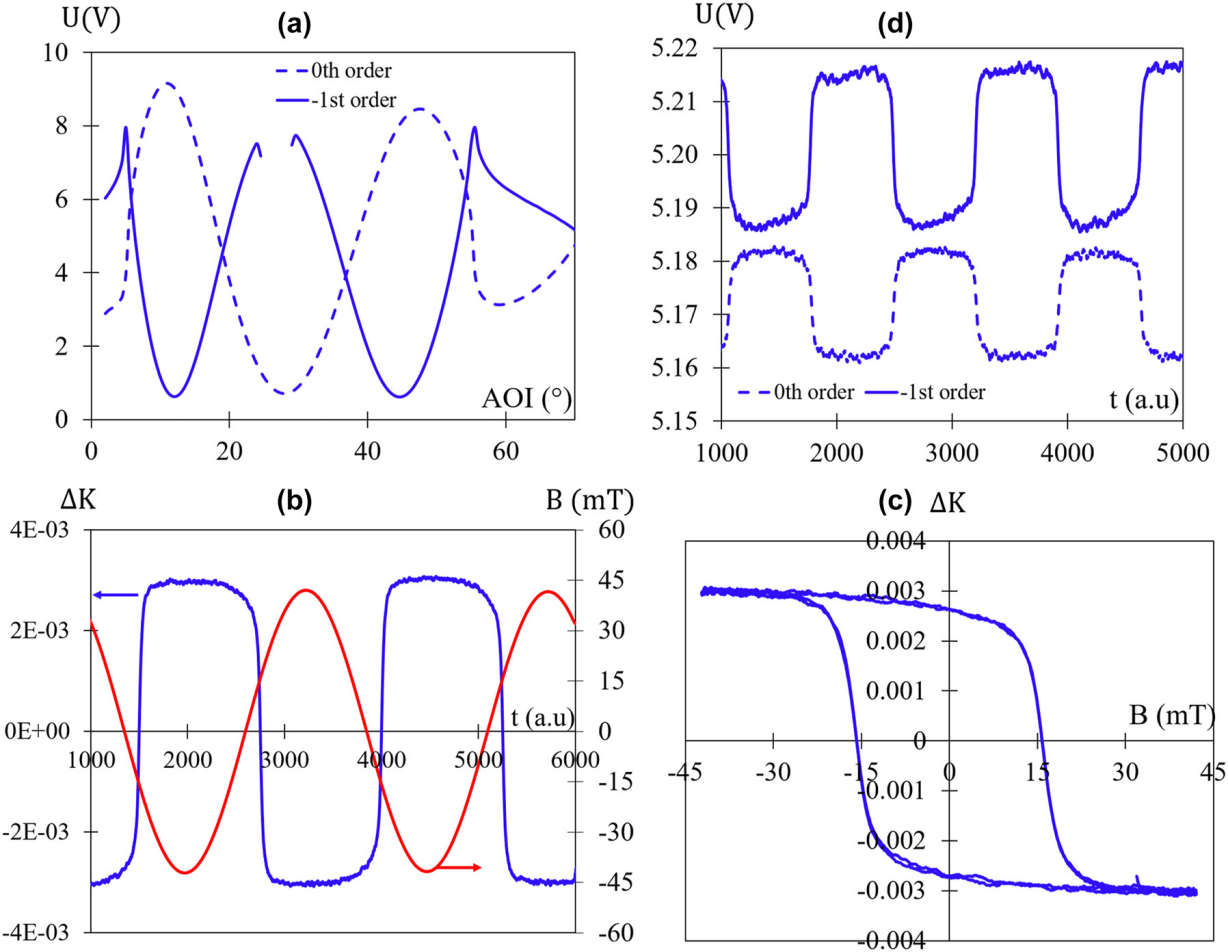
Experimental data. (a) Measured angular spectra of reflected voltage for the 0th and −1st diffracted orders of the magneto-plasmonic structure with 8 nm thickness of Co. The incident light is TM polarization. (b) Time evolution of the normalized voltage difference Δ*K* (blue curve) under a sinusoidal magnetic field (red curve) with a frequency (*F*) of 200 mHz. Δ*K* is defined by Eq. (2). (c) Hysteresis loop of Δ*K* with the data of (b). (d) Measured time evolution of reflected voltage for 0th and −1st diffracted orders when the sinusoidal magnetic field of (b) is applied. All these curves pertain to a Au-grating with 8 nm of Co deposited on top. (b)–(d) correspond to an AOI = 37° (second crossing point of (a)).

The measurement procedure involves continuous measurement of the variation of the voltage related to the 0th and −1st diffraction orders as a function of the applied magnetic field. The angle of incidence is fixed at the crossing points value, at zero field. Subsequently, the normalized voltage difference (Δ*K*) is plotted as a function of the magnetic field (see Figure 4(c)).

(2) $$\Delta K=\frac{U_{0th}-U_{-1st}}{U_{0th}+U_{-1st}}$$

Therefore, when there is no magnetic field applied, Δ*K* remains at zero (crossing point), and it varies with the magnetic field until the magnetization of the film reaches the saturation.

## 4 Results and discussions

Figure 4(a) illustrates the measured reflected voltage angular spectra for the grating with 8 nm of Co for the two orders (0 and −1). As seen in this figure, the second crossing point is around 37°. This value is lower than that presented in the numerical simulations of Figure 1(c) (blue curves), and this difference may be attributed to the surface roughness of the structure. For a more in-depth understanding of the impact of roughness on the switch behavior, readers are encouraged to refer to reference [25].

Figure 4(b) illustrates the normalized voltage difference (Δ*K*) for the grating with an 8 nm Co film at an angle of incidence (AOI) of 37° when the magnetic field undergoes sinusoidal variations (frequency, *F* = 200 mHz). As shown in this figure, the application of a magnetic field leads to a noticeable modification in Δ*K*, and this effect is non-reciprocal concerning the magnetic field. This non-reciprocity results in a hysteresis loop, as depicted in Figure 4(c). In the absence of a magnetic field, the difference (Δ*K* = 0) may initially appear close to zero, which might seem consistent with Figure 4(a). However, this apparent ‘zero’ value is influenced by the notable presence of a large remanent MO effect.

It’s noteworthy that the behavior of the two orders’ voltages under a magnetic field is opposite (see Figure 4(d)): when one (*U*_0th_) increases, the other one (*U*_−1st_) decreases, and *vice versa*. This behavior fits well with the simulated curves in Figure 1(e). The measured MO effect, represented as the peak-to-peak Δ*K* in Figure 4(b), is 0.6 % for a saturation magnetic field of 40 mT. This value is very close to the simulated one (Figure 1(e)).

It is important to note that these differential measurements effectively eliminate common drift and perturbations that could affect both orders. This aspect makes this technology highly promising compared to other MO-SPR sensors [15], [16], which operate with the typical reflection dip resulting from single-order plasmon coupling.

**Figure 5:**
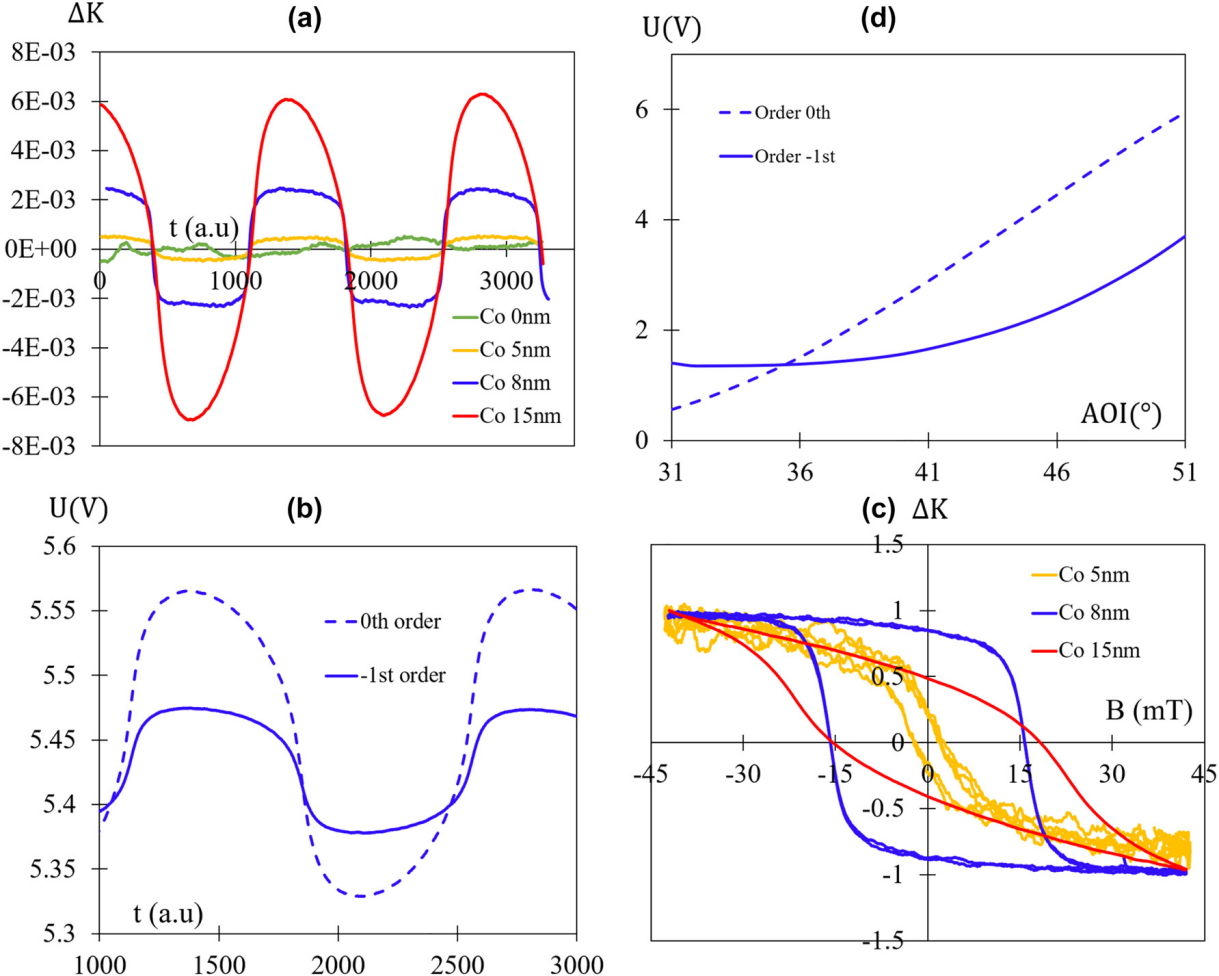
Effect of Co thickness. (a) Normalized voltage difference (Δ*K*) for different Co thicknesses when a sinusoidal magnetic field is applied (*F* = 700 mHz) and at AOI corresponding to the second crossing point of each switch optical curve. (b) Time evolution of the reflected voltage of the 0th and −1st diffracted orders of the structure with 15 nm of Co under the applied magnetic field and an AOI = 35.5°. (c) Hysteresis loop of Δ*K* for different Co thicknesses. (d) Measured optical switch, without magnetic field, for the structure with 15 nm of Co.

Figure 5(a) shows the evolution of Δ*K* for various Co thicknesses under a sinusoidal magnetic field with a frequency (*F*) of 700 mHz. Firstly, a minor derivative signal is observed for the grating without a Co layer (green curve), possibly due to the thermal heating of the coils. Secondly, the effect increases with Co thicknesses. The effect (Δ*K*) undergoes a one-order-of-magnitude increment (Δ*K* = 0.006 peak-to-peak) for the grating with an 8 nm Co film compared to that with 5 nm (Δ*K* = 0.0004 peak-to-peak) and reaches a high value for 15 nm (Δ*K* = 0.013 peak-to-peak). Finally, the effect (for 15 nm) is not saturated at 40 mT, and the behavior of the two orders is not opposite (see Figure 5(b)). Consequently, in the case of 15 nm, the effect of the −1st order is higher (2 % peak-to-peak) than the differential measurements, but the latter is more effective at canceling the measurement noise of both orders. This behavior aligns with the optical switch curve (Figure 4(d)), which reveals that the slopes of both orders are not opposite.

Figure 5(c) shows the normalized hysteresis loops for different thicknesses of Co. It is evident that the coercive field (*B*_*c*_) significantly decreases by an order of magnitude (*B*_*c*_ = 2 mT) for the grating with a 5 nm Co film compared to the one with an 8 nm Co film (*B*_*c*_ = 16 mT). However, the coercive field for the 15 nm Co film must be higher than depicted in Figure 5(c). This is because the hysteresis loop measuring the effect is not saturated at 40 mT, indicating a minor loop with a lower coercive field. The square-like hysteresis loop observed in the 8 nm Co film is absent for both 5 nm and 15 nm thicknesses. This observation aligns with findings in a study by Vahaplar et al. [26].

Therefore, the selection of a 5 nm thickness for the Co layer is preferable since it represents a good balance between maintaining a low-loss structure (crucial for optical switching) and ensuring the closest linearity of magnetic behavior compared to the other thicknesses. These factors are essential for an effective magnetic field sensor.

The choice of ferromagnetic material is particularly relevant, as it directly impacts aspects such as hysteresis, linearity/non-linearity of the response, and the range of the magnetic field that the device can accurately detect. Co was chosen for its simplicity and as a well-known material used for proof of concept. Nevertheless, future studies will explore superparamagnetic materials, which exhibit smaller hysteresis loops and more linear behavior with the magnetic field, aiming to increase the detection limit of the sensor.

## 5 Conclusions

Magnetic field detection is demonstrated numerically and experimentally in this paper using a cost-effective magneto-plasmonic “switch” device. This structure consists of a thin cobalt layer deposited on top of a deep gold grating. Unlike the typical reflection dip resulting from the high-loss single-order plasmon coupling, the reflection angular spectra of this grating significantly differ. It consists of two low-loss orders (0th and −1st) ensuring differential measurement. When a transverse magnetic field is applied to the structure, a high-contrast switching is measured between these two orders: up to 1.3 % with 15 nm Co film. Therefore, this technique is specifically designed to eliminate variations in source power and environmental noise, promising significant applications in magnetic field detection.

## References

[1] Meneghello A., Sonato A., Ruffato G., Zacco G., Romanato F. (2017). A novel high sensitive surface plasmon resonance Legionella pneumophila sensing platform. *Sens. Actuators, B*.

[2] Wong W. R., Fan H., Adikan F. R. M., Berini P. (2018). Multichannel long-range surface plasmon waveguides for parallel biosensing. *J. Lightwave Technol.*.

[3] Sadeghi Z., Shirkani H. (2020). Highly sensitive mid-infrared SPR biosensor for a wide range of biomolecules and biological cells based on graphene-gold grating. *Phys. E*.

[4] Zhang C., Liu Z., Cai C., Yang Z., Qi Z.-M. (2022). Surface plasmon resonance gas sensor with a nanoporous gold film. *Opt. Lett.*.

[5] Maier S. A., Maier S. A. (2007). Excitation of surface plasmon Polaritons at planar interfaces. *Plasmonics: Fundamentals and Applications*.

[6] Rizal C., Belotelov V., Ignatyeva D., Zvezdin A. K., Pisana S. (2019). Surface plasmon resonance (SPR) to magneto-optic SPR. *Condens. Matter*.

[7] Slavík R., Homola J., Brynda E. (2002). A miniature fiber optic surface plasmon resonance sensor for fast detection of Staphylococcal enterotoxin B. *Biosens. Bioelectron.*.

[8] Kodoyianni V. (2011). Label-free analysis of biomolecular interactions using SPR imaging. *BioTechniques*.

[9] Sepúlveda B., Calle A., Lechuga L. M., Armelles G. (2006). Highly sensitive detection of biomolecules with the magneto-optic surface-plasmon-resonance sensor. *Opt. Lett.*.

[10] Zvezdin Kotov A. K. (1997). *V. A. Modern Magnetooptics and Magnetooptical Materials*.

[11] Bsawmaii L., Gamet E., Royer F., Neveu S., Jamon D. (2020). Longitudinal magneto-optical effect enhancement with high transmission through a 1D all-dielectric resonant guided mode grating. *Opt. Express*.

[12] Bsawmaii L., Gamet E., Neveu S., Jamon D., Royer F. (2022). Magnetic nanocomposite films with photo-patterned 1D grating on top enable giant magneto-optical intensity effects. *Opt. Mater. Express*.

[13] Belotelov V. I. (2011). Enhanced magneto-optical effects in magnetoplasmonic crystals. *Nat. Nanotechnol.*.

[14] Wu W. (2019). Compact magnetic field sensor based on a magnetic-fluid-integrated fiber interferometer. *IEEE Magn. Lett.*.

[15] Dong J. (2022). Design and analysis of surface plasmon resonance sensor based on multi-core photonic crystal fiber. *Optik*.

[16] Liu Z., Wang Y., Zhang C. (2023). Magnetic field sensor based on magnetic optical surface plasmon resonance. *Adv. Photonics Res.*.

[17] Tishchenko A., Parriaux O. (2015). Coupled-mode analysis of the low-loss plasmon-triggered switching between the 0 Th and -1 St orders of A metal grating. *Photonics J. IEEE*.

[18] Sauvage-Vincent J. (2013). *Les modes de plasmon sur film métallique ondulé, appliqués aux documents de sécurité*.

[19] Sauvage-Vincent J., Jourlin Y., Petiton V., Tishchenko A. V., Verrier I., Parriaux O. (2014). Low-loss plasmon-triggered switching between reflected free-space diffraction orders. *Opt. Express*.

[20] Popov L. T. E., Maystre D. (1990). Gratings—general properties of the Littrow mounting and energy flow distribution. *J. Mod. Opt.*.

[21] Laffont E., Crespo-Monteiro N., Valour A., Berini P., Jourlin Y. (2023). Differential sensing with replicated plasmonic gratings interrogated in the optical switch configuration. *Sensors*.

[22] Lyndin N. (2013). ..

[23] Jean C., Raoult G., Maystre D. (1980). A new theoretical method for diffraction gratings and its numerical application. *J. Opt.*.

[24] Moharam M. G., Gaylord T. K. (1981). Rigorous coupled-wave analysis of planar-grating diffraction. *J. Opt. Soc. Am. A*.

[25] Bruhier H. (2022). Effect of roughness on surface plasmons propagation along deep and shallow metallic diffraction gratings. *Opt. Lett.*.

[26] Yadagiri K., Wu T. (2020). The thickness of buffer layer and temperature dependent magneto dynamic properties of Ta/FeGaB/Ta tri-layer. *J. Magn. Magn. Mater.*.

